# The Making of a CYP3A Biomarker Panel for Guiding Drug Therapy 

**DOI:** 10.3390/jpm2040175

**Published:** 2012-10-29

**Authors:** Danxin Wang, Wolfgang Sadee

**Affiliations:** Program in Pharmacogenomics, Department of Pharmacology, College of Medicine, The Ohio State University, Columbus, OH 43210, USA; E-Mail: Wolfgang.sadee@osumc.edu

**Keywords:** cytochrome P450s, CYP3A4, polymorphism, biomarker

## Abstract

CYP3A ranks among the most abundant cytochrome P450 enzymes in the liver, playing a dominant role in metabolic elimination of clinically used drugs. A main member in CYP3A family, CYP3A4 expression and activity vary considerably among individuals, attributable to genetic and non-genetic factors, affecting drug dosage and efficacy. However, the extent of genetic influence has remained unclear. This review assesses current knowledge on the genetic factors influencing CYP3A4 activity. Coding region *CYP3A4* polymorphisms are rare and account for only a small portion of inter-person variability in CYP3A metabolism. Except for the promoter allele CYP3A4*1B with ambiguous effect on expression, common *CYP3A4* regulatory polymorphisms were thought to be lacking. Recent studies have identified a relatively common regulatory polymorphism, designated CYP3A4*22 with robust effects on hepatic CYP3A4 expression. Combining CYP3A4*22 with *CYP3A5* alleles *1, *3 and *7 has promise as a biomarker predicting overall CYP3A activity. Also contributing to variable expression, the role of polymorphisms in transcription factors and microRNAs is discussed.

## 1. Introduction

Inter-individual variability in drug absorption, distribution, metabolism and elimination (ADME), arising from both genetic and non-genetic factors, is a main cause of therapeutic failure or undesirable adverse effects. To optimize drug response, multiple strategies have been developed to tailor individual drug therapy, employing pharmacokinetic parameters based methods [1,2,3], pharmacodynamic monitoring [4], toxicity based titration [5], and use of genetic biomarker tests [6]. Genetic variants in *CYP2C9*, *CYP2C19* and *CYP2D6* are already clinically employed for predicting dosages and response for warfarin, clopidogrel, and many CYP2D6 substrate drugs (see FDA Table of Pharmacogenomic Biomarkers in Drug Labels [7]). While CYP3A isozymes, the most abundant and important drug metabolizing enzymes in the liver, display large inter-individual variability [8], valid genetic biomarkers for overall CYP3A activity have yet to be established. As pharmacogenomic biomarkers begin to play a prominent role in personalized medicine, predictive tests for CYP3A activity, in particular that of CYP3A4 and CYP3A5 could have substantial clinical utility. 

**Figure 1:**
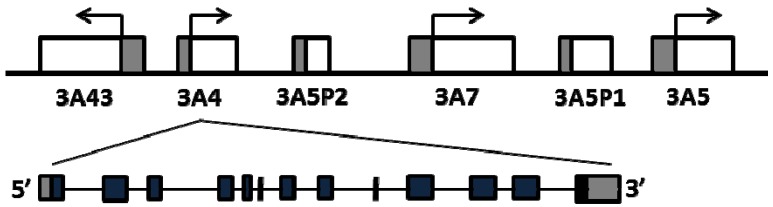
Schematics of the *CYP3A* locus and genomic structure of *CYP3A4*.

The four human *CYP3A* genes cluster on chromosome 7q21 in the order of *CYP3A43*, *CYP3A4*, *CYP3A7* and *CYP3A5* [9,10] (Figure 1). CYP3A4 is the most abundant isoform in liver and intestine, followed by CYP3A5 in individuals with CYP3A5*1/*1 genotype [11]. CYP3A7 is highly and variably expressed in fetal livers, accounting for up to 50% of total cytochrome P450s [12], while the expression level of CYP3A7 decreases rapidly after birth and becomes undetectable in most of adult livers, except for individuals who carry two promoter variants, CYP3A7*1B and CYP3A71C [13]. CYP3A43 is expressed at very low levels in adult livers, accounting for only 0.1%–0.2% CYP3A4 transcripts [10,14]. CYP3A4 and CYP3A5 have similar substrate specificity [15], while CYP3A7 has a smaller substrate spectrum whereas CYP3A43 does not appear to contribute substantially in drug metabolism [16,17]. Considerable inter-person variability in CYP3A expression and enzyme activity has been attributed to genetic and non-genetic factors [18,19,20,21]. While each CYP3A member may contribute to overall CYP3A enzyme variability, CYP3A4 accounts for a majority the hepatic CYP3A activity in most individuals, followed by CYP3A5 [22]. Moreover, CYP3A4 has higher specific activity towards common CYP3A drug substrates than the other isozymes [16]. In the case of CYP3A5 and CYP3A7, known *CYP3A5* alleles (e.g., *3 and *7) and *CYP3A7* (*1B and *1C) largely account for variable expression in adult livers with clinical implications [23]. However, despite large CYP3A4 variability, frequent polymorphisms affecting CYP3A4 activity were thought to be absent, except for the recently described *22 allele. Because the impact of polymorphisms in CYP3A5 and CYP3A7 on drug metabolism depends on the concomitant expression status of CYP3A4, variants in *CYP3A4* or in genes regulating CYP3A4 expression must be considered in developing a genetic biomarker panel for predicting CYP3A activity. Overlaid onto such a genetic allele panel must be an assessment of relative substrate selectivity for each dug and isozymes; in some instances, a drug could be predominantly metabolized by CYP3A5 or CYP3A7 so that CYP3A4 activity then has less impact. 

This review focuses on factors that determine variable CYP3A4 expression/enzyme activity, including common regulatory polymorphisms and rare coding region *CYP3A4* polymorphisms, interaction between *CYP3A4* and *CYP3A5* alleles, and *trans*-acting factors that regulate CYP3A4 expression. We also discuss the clinical utilities of these factors as biomarkers for predicting CYP3A activity. Because of the eminent role of CYP3A4 in drug metabolism, we discuss in detail all proposed functional variants; even if they are rare, as new genotyping and sequencing methodologies enable rare variants with clear evidence for effect on expressed protein activity to be incorporated into predictive biomarker panels.

## 2. Common Regulatory Polymorphisms in *CYP3A4*

Previous studies have focused on promoter SNP rs2740574 (CYP3A4 *1B, −392A>G, MAF 5.4% in Caucasians and 35% in non-Caucasians) [19,24,25]. Despite numerous clinical association studies, the function of CYP3A4*1B remains controversial [19,23,26,27,28,29,30]. Moreover, CYP3A4*1B is in LD with the fully active CYP3A5*1 reference allele in African Americans [31,32], raising the possibility that CYP3A5 activity could have accounted for any clinical phenotype associated with CYP3A4 *1B. 

Proposed additional regulatory variants reside in intron 7 (SNP rs4646437), intron 10 (SNP rs2242480), and in putative enhancer regions. Intron 7 SNP rs4646437 was associated with CYP3A4 protein expression and enzyme activity in human liver microsomes, a finding observed only in males but not in females [33]; absent of a convincing mechanism, this result requires further study. The intron 10 SNP CYP3A4*1G (rs2242480) has been associated with lipid-lowering efficacy of atorvastatin [34], tacrolimus pharmacokinetics in renal transplant patients [35], risk of coronary heart disease [36] and severity of withdrawal symptoms in methadone maintenance patients [37], all studies performed in Asian populations. However, the effect of CYP3A4*1G on mRNA/protein level remains uncertain. In reporter gene assays, the minor *G* allele resulted in reduced transcription [36], suggesting a loss of function. However, the *G* allele was associated with lower dose-adjusted blood levels (AUC) of tacrolimus *in vivo* [35], implicating a gain of function (although reporter gene assays in a heterologous tissue may not be predictive of *in vivo* effects). Again, CYP3A4*1G is in high LD with CYP3A5 *1 in Japanese individuals [35,38], confounding the analysis. Variants in the enhancer region of *CYP3A4* include rs2737418 (−7,206 upstream), found to be associated with CYP3A4 enzyme activity in livers from African Americans, but the results for enzyme activity and mRNA levels were contradictory, the minor T allele being associated with higher enzyme activity but lower mRNA levels [39]. Similarly, while reporter gene assays suggested an effect for a *TGT* insertion (rs34401238, −11 kb upstream) on *CYP3A4* transcription, any *in vivo* effect remains unknown [40]. Moreover, allelic RNA expression in results in human livers argues against a detectable effect on RNA expression for any of these regulatory SNPs mentioned above [41]. The inconsistent clinical associations observed with these variants may be caused in part by LD with CYP3A5*1, as suggested by a recent study [42].

Recently, we have identified an intron 6 SNP (rs35599367, designated as *22) in *CYP3A4* that strongly affects hepatic expression and is associated with statin dose requirements [41]. Measuring allelic CYP3A4 heteronuclear RNA (hnRNA) and mRNA expression in human livers using multiple marker SNPs, we found marked allelic expression imbalance in 13% of samples. Genotyping and sequencing the *CYP3A4* locus identified intron 6 SNP *rs35599367*, which fully accounted for the allelic CYP3A4 expression imbalance; with the minor *T* allele expressed 1.6–5 folds less than the main *C* allele. Consistently, CYP3A4 mRNA and enzyme activity in livers with CC genotype were higher than CT and TT carriers. While the underlying molecular mechanism remains unclear, *in vitro* minigene transfection assays suggested that the *T* allele affect nascent RNA elongation [41]. Consistent with reduced enzyme activity supported by CYP3A4*22, others have reported that *22 was associated with tacrolimus pharmacokinetics in kidney transplant recipients [43], cyclosporine and tacrolimus trough blood levels and dose requirements [44], simvastatin-mediated cholesterol reduction [45], and increased risk of delayed graft function and worse renal function in cyclosporine-treated kidney transplant patients [46]. The allele frequency of *22 is 3%–6% in Caucasian population and may be lower in subjects of African descent. Because CYP3A4 metabolizes nearly 50% of clinically used drugs, *22 could become a broadly useful biomarker to predict drug dosage, efficacy, and adverse effects. As CYP3A4*22 is embedded in main reference haplotype, lacking substantial LD with any other HapMap SNPs, it had escaped detection in genome-wide association studies built on tagging SNP panels.

## 3. Coding Region Polymorphisms

More than 20 non-synonymous coding region SNPs have been reported for *CYP3A4* with low minor allele frequency (MAF) for each (Table 1). A critical analysis of these variants is essential for future biomarker panel development.

CYP3A4 *2, *3, *7, *9, *10, *16, *17 and *19 support similar protein expression compared to the reference allele *1, measured in a bacterial expression system. CYP3A4*3, *7, *9, *10 and *19 also did not change enzyme activity, while CYP3A4 *2, *7, *16 and *17 caused substrate specific changes in enzyme activity [25,47,48,49] CYP3A4*3 was associated with lower levels of low-density lipoprotein cholesterol in hypercholesterolemia patients but lacking a cogent mechanistic interpretation [50], and with a higher HDL increase upon fluvastatin treatment [51]. On the other hand, CYP3A4*3 was not associated with changes in LDL level after atorvastatin treatment [50,51], a CYP3A4 substrate, arguing against a substantial effect on enzyme activity and hence drug effect. In Japanese cancer patients receiving paclitaxel, *1/*16 carriers showed a 20% lower median AUC ratio of 3'-p-hydroxypaclitaxel to paclitaxel and a 2.4 fold higher median AUC ratio of 6α-hydroxypaclitaxel to paclitaxel, compared to *1/*1 carriers, indicating *16 is associated with reduced 3'-p-hydroxylation. of paclitaxel [52]. This finding requires further study.

**Table 1:** Coding region SNPs in CYP3A4.

Allele	variant (cDNA)	Amino acid change	Key SNP rs#	Gene location	MAF				*In vitro* effect	*In vivo* effect	Ref
					Caucasian	African American	Asian	All (ESP)			
*2	664 T>C	S222P	rs55785340	Exon 7	0.027	0	0		Decreased activity		[44]
*3	1334 T>C	M445T	rs4986910	Exon 12	0–0.04	0–0.033	0		No change		[43,45,46,47]
*4	352 A>G	I118V	rs55951658	Exon 5			0.015–0.033			Decreased activity, associated with LDL	[50,51]
*5	653 C>G	P218R	rs55901263	Exon 7	0	0	0.006			Decreased activity	[50]
*6	830_831 insA	277 frameshift	rs4646438	Exon 9			0.005			No activity	[50]
*7	167 G>A	G56D	rs56324128	Exon 3	0.014				Decreased activity		[43,44]
*8	389 G>A	R130Q	rs72552799	Exon 5	0.0033				No protein		[43]
*9	508 G>A	V170I	rs72552798	Exon 6	0.0024				No change		[43]
*10	520 G>C or G>A	D174H or D174N	rs4986908	Exon 6	0.003 for C		0.012 for A		No change		[21,43]
*11	1088 C>T	T363M	rs67784355	Exon 11	0.0034		0.002	0.001	Decreased protein level and activity		[43,49]
*12	I117 C>T	L373F	rs12721629	Exon 11	0–0.004	0.004–0.007	0	0.001	Decreased protein level and activity		[43]
*13	1247 C>T	P416L	rs4986909	Exon 11	0–0.004	0–0.021	0–0.012		No protein		[43]
*14	44 T>C	L15P	rs12721634	Exon 1				0–0.003			[21]
*15	485 G>A	R162Q	rs4986907	Exon 6	0	0–0.042	0	0.014			[21]
*16	554 C>G	T185S	rs12721627	Exon 7			0.014	0.005	Decreased protein level and activity		[44,48,49]
*17	566 T>C	F189S	rs4987161	Exon 7	0–0.017	0	0		Decreased activity		[45]
*18	878 T>C	L293P	rs28371759	Exon 10			0.028	0.01	No change or increased activity	Low midazolam clearance, associated with bone density	[44,45,48,49,52,53,54,55]
*19	1399 C>T	P467S	rs4986913	Exon 12	0–0.022	0	0–0.012		No change		[45]
*20	1461_1462 insA	488 frameshift	rs7666821	Exon 13	<0.006				No activity	Low midazolam clearance	[56]
*21	956 A>G	Y319C		Exon 10			0.005				[21]

CYP3A4*8 and *13 did not yield detectable P450 holoproteins in a bacterial expression system, indicating they may affect protein translation or protein stability [47]. Similarly, CYP3A4*11 and *12 showed less protein expression than *1 in *in vitro* expression system, while CYP3A4*12 also altered substrate-specific enzyme activity [47,53]. No *in vivo* effects or association studies have been reported, owing to the low allele frequencies of these variants; therefore, it is premature to consider these alleles in clinical biomarker panels until more results are available, specifically on *8 and *13 expression in human cells.

CYP3A4 *4, *5 and *6 occur only in Asian populations with MAF of 1.5%–3%, <1%, and <1%, respectively. Subjects heterozygous for *4, *5, and *6 displayed decreased urinary ratios of 6β-hydroxycortisol over cortisol as a measure of CYP3A4 activity, but the number of subjects was small and all were taking additional drugs such as ketoconazole [54], shedding doubt over any *in vivo* effect. In addition, after 4 weeks of oral administration of 20 mg simvastatin, *4 carriers had greater reduction in triglycerides and total cholesterol than non-carriers [55], but again relying on a very small sample size. As CYP3A4*6 represents an *A* insertion causing a frameshift, it is likely to abolish CYP3A4 enzyme activity.

CYP3A4*15 exists only in African Americans with MAF of 4.2%. One study found that individuals with *15 also express CYP3A5 [25], indicating *15 may be in LD with CYP3A5 *1 [25]. On the other hand, CYP3A4*18 is specific to Asian population with MAF of 2.8%. *In vitro* expression results indicate that *18 expresses similar protein levels as *1 and did not alter catalytic activity [48,49,53]. Yet, in 13 subjects heterozygous for *18 and 26 control subjects, *18 carriers showed diminished midazolam clearance compared to non-carriers (and also with low lumbar spine bone mass density) [56], suggesting *18 is associated with decreased catalytic activity for midazolam. Molecular modeling suggested structural change in substrate recognition sites in *18 compared to *1. In contrast, *18 was associated with decreased Cmax and AUC, and increased clearance of cyclosporine in healthy Chinese subjects, suggesting higher enzymatic activity [57]. Similarly, *18 was associated with increase clearance of tacrolimus in healthy Chinese subjects [58], and in renal transplant recipients [59]. Yet, in Japanese cancer patients, *18 was not associated with paclitaxel pharmacokinetic parameters [52]. These results are compatible with the hypothesis that *18 has substrate-specific properties, but more direct evidence is needed to test the validity of this mechanism. In a cohort of 2125 Korean women, *18 was in high linkage disequilibrium (LD) with CYP3A5*3 (D' = 0.8). As discussed earlier, high LD between haplotypes spanning *CYP3A4 and CYP3A5* confounds conclusion about CYP3A4 activity by effects on CYP3A5. 

CYP3A4*20 has a MAF of <0.6% in Caucasian population. Representing an A insertion, *20 shifts the open reading frame and creates a premature stop codon yielding a truncated protein. In yeast and HEK cell expression system, *20 showed low protein expression lacking catalytic activity for midazolam. One subject heterozygous for *20 showed reduced systemic midazolam clearance [60]. We surmise that the evidence for *20 abolishing CYP3A4 activity is strong.

In summary, the MAF’s of coding region SNPs are exceedingly low. Non-synonymous SNPs with MAF >1% either did not change activity/protein expression or their functions have not been fully characterized (*2, *3, *7, *9, *10, *15, *17, *19). CYP3A4 *4, *5, *6, *8, *11, *12, *13, *16 and *20 appear to decrease protein expression and/or enzyme activity, but display MAF’s <1%, mostly in only one ethnic group, and with varying degree of evidence supported a robust effect on CYP3A4 activity. However, anticipating the arrival of large scale sequencing in clinical practice, it now becomes imperative to assess the impact of variants with low minor allele frequency, as these will become available when full sequences are being deployed. Some rare coding region variants (for example, SNPs that shift open reading frame or encode an unstable protein like *6, *20, *8, *11, *12 and *13) may have significant impact on drug metabolism in individuals who carry them and may become valuable biomarkers for predicting CYP3A activity in comprehensive biomarker panels. In Table 2, we present those alleles with currently the strongest evidence of functional variation, while additional studies are needed to validate them for clinical utility.

**Table 2:** Mutations in *CYP3A4* and *CYP3A5* as potential biomarkers for predicting CYP3A activity.

Gene	Allele	Location, variant	MAF	dbSNP number	CYP3A4 activity
*CYP3A4*	*1 ^a^	-	-	-	-
*CYP3A4*	*22	Intron 6, C>T	3%–6%	rs35599367	Decreased protein
*CYP3A4*	*6 ^b^	Exon 9, insA	rare	rs4646438	Frameshift
*CYP3A4*	*20 ^b^	Exon 13, insA	rare	rs7666821	Frameshift
*CYP3A5*	*1 ^a^	-	-	-	-
*CYP3A5*	*3	Intron3, G>A	15%–88%	rs776746	No protein
*CYP3A5*	*7	Exon 11, delT	4%–21%	rs41303343	Frameshift

^a^ Reference allele; ^b^ Rare variants to be considered with large-scale parallel genotyping/sequencing in clinical practice.

## 4. Interaction between CYP3A4 and CYP3A5

Because drugs metabolized by CYP3A4 are often also CYP3A5 substrates while CYP3A5 expression is comparable to that of CYP3A4 in some individuals [11], CYP3A5 could contribute substantially to inter-individual variability in CYP3A activity. Therefore, the interaction between *CYP3A4* and *CYP3A5* polymorphisms has to be considered. The most frequent polymorphism in *CYP3A5* (*3, rs776746), causing aberrant splicing and abolishing CYP3A5 mRNA and enzyme activity [11,61] (Table 2), is highly prevalent in Caucasians (>80%) but less frequent in African Americans (<20%). Since CYP3A4*22 and CYP3A5*3 are in very low LD, loss of CYP3A function caused by *22 can be compensated by CYP3A5 expression in individual carrying the wild-type CYP3A5*1 allele. CYP3A5*3 has been associated with tacrolimus/cyclosporine pharmacokinetics, but the results are not always consistent [62], especially for cyclosporine. Because tacrolimus is preferentially metabolized by CYP3A5, and cyclosporine by CYP3A4, it is possible that the inconsistent associations were caused by neglecting the activity and functional polymorphism in CYP3A4. With discovery of the relatively common functional polymorphisms *22 in *CYP3A4*, it is now possible to test the *in vivo* effects of the combination of *CYP3A4* and *CYP3A5* alleles on CYP3A activity. For example, the association between *22 and simvastatin-mediated cholesterol reduction is stronger when analyzed only in individuals who carry CYP3A5*3/*3 than in the entire cohort [45]. Similarly, the association between *22 and tacrolimus pharmacokinetics and tacrolimus/cyclosporine dose requirements became stronger when CYP3A5 *3 genotype was considered [43,44]. Moreover, CYP3A7 variants CYP3A7*1B and CYP3A7*1C may also contribute to inter-person variability in CYP3A activity for some substrates especially steroids in adult livers [23]. However, the impact of CYP3A7 in clinical drug metabolism is still largely unknown. 

## 5. Polymorphisms in Transcription Factors

CYP3A4 is subject to regulation by several transcription factors, including liver enriched transcription factors and nuclear receptor family [63,64,65,66]. The expression of CYP3A4 mRNA is correlated with the expression of PXR, CAR, HNF4α, FOXA2, and others [41,67,68]. Therefore, polymorphisms present in these transcription factors could regulate the constitutive and inducible expression of CYP3A4. However, coding region SNPs in PXR and CAR are rare, and any effect on CYP3A4 expression is unclear [69]. Common polymorphisms in promoter, intron or downstream regions have been identified in PXR, some of them significantly associated with CYP3A4 mRNA expression or enzyme activity [70,71]. In one study, rs2472677 has been associated with unboosted atazanavir clearance [72], while a *PXR* haplotype was found to be associated with CYP3A4 and ABCB1 mRNA expression and doxorubicin clearance in Asian breast cancer patients [73]. Yet, further results failed to confirm associations between *PXR*, *CAR* and *HNF4α* genotype and docetaxel/ doxorubicin pharmacokinetics [74,75]. These studies suggest that polymorphisms in transcription factors have the potential to affect CYP3A4 expression, but likely account only for a portion of inter-individual variability in CYP3A4 expression and enzyme activity, being confounded by environmental conditions that impact multiple transcription factor expression. However, the polymorphisms identified by associations often lack a clear mechanistic role and likely represent only tagging SNPs, so that the effect size of true regulatory variants remains unclear. In addition, PXR, CAR and other transcriptions factors are subject to alternative splicing, with splice variants having different activities in DNA binding or ligand binding [69,70,76,77]. It is unknown whether splicing events are regulated by *cis*-acting polymorphisms; therefore, any genetic *trans-*acting effect on CYP3A enzymes remains open for study. At present, genetic variants in transcription factors can therefore not be employed in clinical biomarker panels predictive of CYP3A activity.

## 6. microRNA and Epigenetics Regulation

CYP3A4 expression is further regulated directly or indirectly by miRNAs. miR-27b binds to a *CYP3A4* 3'UTR target sequence and decreases CYP3A4 mRNA and protein expression, thereby reducing *in vitro* sensitivity of PANC1 cells to cyclophosphamide [78]. Moreover, CYP3A4 expression can be regulated indirectly by miR-148a or miR-27b via targeting PXR [79] or VDR [78]. Furthermore, CYP3A4 expression appears to be regulated by epigenetic modifications directly or indirectly through transcription factor PXR [80,81]. These regulatory events may contribute to the large inter-individual variability of hepatic CYP3A4 but cannot be readily translated into a biomarker test.

## 7. Clinical Implications

Although coding region polymorphisms of CYP3A4 are included in the Affymetrix drug-metabolizing enzymes and transporters panel (DMET plus), they usually are not detected because of MAF < 1% in Caucasian or Caucasian/African American mixed populations [42]. Therefore, their impact on clinical phenotypes can be assessed only by inference from *in vitro* studies, unless very large cohorts are studied—limiting their clinical utility. CYP3A4*18 is relatively frequent in Asian population, showing some clinical associations, but its function on enzyme activity is controversial and the evidence insufficient for clinical decision making [48,49,53,56,57,58]. Also, because CYP3A4*18 is in LD with CYP3A5*1, the independent effect of CYP3A4*18 may be limited [56]. CYP3A5 *3 was proposed to be a biomarker for predicting tacrolimus dose in combination with clinical factors [82,83], but a significant portion of observed inter-individual variability remains unaccounted for [84,85], possibly caused by neglecting CYP3A4 polymorphisms or using previously proposed regulatory *CYP3A4* SNPs relying on ambiguous results [86,87,88]. On the other hand, the recently discovered regulatory CYP3A4*22 allele (rs35599367) [41] has promise as a useful biomarker for predicting CYP3A4 enzyme activity. Combined with *CYP3A5* genotype (*1, *3 and *7), it is now possible to categorize individuals into poor, intermediate and extensive metabolizer phenotypes for overall CYP3A activity [43]. Indeed, a combination of *CYP3A4* and *CYP3A5* genotypes predicted tacrolimus/cyclosporine dose or pharmacokinetics parameters better than *CYP3A4* or *CYP3A5* genotype alone [43,44]. Although CYP3A4 expression is further regulated by polymorphisms in transcription factors and the expression of microRNAs, their clinical utility is unknown at the present. 

In conclusion, multiple factors regulate CYP3A4 expression/enzyme activity. Yet, at this point only one relatively common regulatory polymorphism rs35599367 (CYP3A4*22) has promise as a biomarker. While the *22 allele contributes a relatively small portion to overall population variance in CYP3A4 activity, owing to its relatively low allele frequency, it does have significant influence on those subjects carrying the minor allele. Homozygous carriers of *22 may be rare (<<1%), but given the large number of subjects taking CYP3A4 substrate drugs, such individuals may experience unusual drug effects. Combined with well-established variants in *CYP3A5* (*3 and *7), it is now possible to predict a larger portion of the genetic components contributing to overall CYP3A enzyme activity toward CYP3A substrate drugs across different racial groups. Rare mutations with obvious and documented effect on enzyme activity can be incorporated into a predictive panel once clinical genotyping/sequencing is routinely performed on a broader scale. 
